# Recurrent Fetal Loss and Antiphospholipid Antibodies: Clinical and Therapeutic Aspects

**DOI:** 10.1155/S1064744997000288

**Published:** 1997

**Authors:** O. Blétry, A.-M. Piette

**Affiliations:** Service de Médecine Interne Hôpital Foch 40 rue Worth Suresnes 92151 France

## Abstract

Recurrent fetal losses indicate screening for antiphospholipid antibodies, especially after the third consecutive fetal loss, or when they occur after 12 weeks gestation or when the mother presents with thrombosis or other ailments of antiphospholipid syndrome. Fetal loss may be caused by thromboses of placental vasculature. There is no agreement concerning the mechanism of thromboses: protein C pathway and/or annexin V are the best candidates. When fetal loss occurs early during gestation, murine models suggest that antiphospholipid antibodies can also act on trophoblasts by inhibiting syncytia formation. Among the high risk patients with more than two fetal losses, an association of aspirin and heparin given early during gestation is successful in 70–80% of cases.


Infectious Diseases in Obstetrics and Gynecology 5:183–191 (1997)
© 1997 Wiley-Liss, Inc.

### Recurrent Fetal Loss and Antiphospholipid Antibodies: Clinical and Therapeutic Aspects

**O. Blétry* and A.-M. Piette**

*Service de Médecine Interne, Hôpital Foch, Suresnes, France*

ABSTRACT

**Recurrent fetal losses indicate screening for antiphospholipid antibodies, especially after the third consecutive fetal loss, or when they occur after 12 weeks gestation or when the mother presents with thrombosis or other ailments of antiphospholipid syndrome. Fetal loss may be caused by thromboses of placental vasculature. There is no agreement concerning the mechanism of thromboses: protein C pathway and/or annexin V are the best candidates. When fetal loss occurs early during gestation, murine models suggest that antiphospholipid antibodies can also act on trophoblasts by inhibiting syncytia formation. Among the high risk patients with more than two fetal losses, an association of aspirin and heparin given early during gestation is successful in 70–80% of cases. Infect. Dis. Obstet. Gynecol. 5:183–191, 1997.** © 1997 Wiley-Liss, Inc.

KEY WORDS

fetal loss; habitual abortion; antiphospholipid syndrome; systemic lupus erythematosus; protein C pathway; annexin V; antithrombotic therapy

Recurrent fetal loss (FL) affects 1–5% of women of childbearing age.[1,2] Chromosomal abnormalities account for 60% of recurrent FL. Immunological mechanisms may account for the remaining 40%, and in particular, the antiphospholipid syndrome (APLS) appears to be implicated in 7–25% of recurrent FL.[2]

In 1980, Soulier and Boffa[3] suggested a relationship between recurrent FL and lupus anticoagulant (LA). The APLS was defined 7 years later[4] based on a combination of clinical and biological abnormalities: on the one hand, venous or arterial thrombosis and/or recurrent FL; on the other, recurrent evidence of LA and/or anticardiolipin antibodies (ACL). Objections may be raised to this definition,[5] one of the major problems being the interlaboratory variation in ACL assays and the absence of consensus concerning the normal upper limit of ACL.[6] In our laboratory, we consider the normal upper limit to be 20 GPL units for IgG ACL and 20 MPL units for IgM ACL.

Antiphospholipid-associated obstetrical disorders are not limited to FL. They also include infertility, intrauterine growth retardation, fetal distress, prematurity,[7] and in the mother, preeclampsia[8] and chorea gravidarum.[9] We shall limit ourselves to FL, a term including abortion and fetal death, the limit between these being considered by Branch[10] to be at 20 weeks gestation.

### WHEN TO SCREEN FOR ANTIPHOSPHOLIPID ANTIBODIES (APL)?

It is generally considered that screening for APL is unnecessary in asymptomatic gravida 0 or nulliparous women.[11–13] Indeed, LA and ACL positivity are infrequent in these women, being present in less than 4%, and the relationship to the outcome of pregnancy is unclear. However, the findings of Lynch et al.[14] are conflicting, with a 24.4% PL positivity rate among nearly 400 nulliparous patients, and a 2.44 relative risk of FL among APL-positive women.

*Correspondence to: O. Blétry, Service de Médecine Interne, Hôpital Foch, 40 rue Worth, 92151 Suresnes, France.

Received 1 October 1997

Accepted 21 October 1997

TABLE 1. APL frequency in FL

| | | Results (%) | |
|---|---|---|---|
| | Author | LA | ACL (IgG) |
| One FL | Infante-Rivard et al., 1991[15] | 3.8 | 1.5 |
| Two FL | Cowchock et al., 1986[16] | | 13 |
| | Edelman et al., 1986[17] | 14 | |
| Three FL | Howard et al., 1987[18] | 48 | |
| | Petri et al., 1987 | 9 | 11 |
| | Unander et al., 1987[19] | | 35 |
| | Creagh et al., 1991[20] | 20 | 17 |
| | Parke et al., 1991[13] | 4.5 | 6.8 |
| | Rai et al., 1955[25] | 9.1 | 3.3 |

Screening for APL also seems unjustified after a first FL. The case-control study conducted by Infante-Rivard et al.[15] with 331 patients and 993 controls showed 5.1% LA positivity in patients vs. 3.8% in controls (relative risk:1.42) and 1.2% IgG ACL positivity in patients vs. 1.5% in controls.

On the other hand, screening becomes cost effective after two, or better, three FL.[13,16–20] The problem is that the percent APL positivity in these patients varies widely in different studies. Table 1 shows an LA variation from 4.5% to 48%, and the percent IgG ACL positivity varies from 6.8% to 35%. There is generally a significant difference between patient and control groups, except in the study by Petri et al.[21] In this study, the difference between a group of 44 patients with recurrent FL and a group of 40 controls was not significant; however, a number of questions can be raised about this study: the sensitivity of the method used for LA testing (using the dilute Russell's viper venom time) is probably too low, and the study population is not large enough to detect a significant difference (9% LA positivity in patients vs. 0% in controls, and 11% ACL positivity in patients vs. 2.5% in controls); moreover, in this study, more than 50% of patients had very early miscarriages.

Apart from the number of FL, other criteria should be taken into account, especially the date of the FL. Within a pregnancy, the first 12 weeks of gestation (the preembryonic and embryonic periods) should be distinguished from the following weeks which are, strictly speaking, the fetal period. According to Branch et al,[22] whose experience of FL is quite extensive; FL occurs within the first 12 weeks of gestation in nearly half of cases, and in the other half, mostly between 16 and 20 weeks gestation. However, in several large studies, especially in a number of recent randomized therapeutic trials, pretreatment FL occurred mostly during the first trimester.[23–25] In Kutteh's[24] study, 80% of pretreatment FL occurred before 12 weeks gestation. In any case, the occurrence of FL after 12 weeks gestation rules out a syndrome of recurrent early pregnancy loss, or intrauterine embryonic death,[1,26] which always occurs before 12 weeks gestation and is not associated with detectable antibodies in the mother, and might be due to antitrophoblast natural killer (NK) cell toxicity.[27]

The inevitable occurrence of FL in all subsequent pregnancies in women who have experienced one FL is also a strong argument for testing for APL. Lubbe and Liggins[28] and Branch et al.[29] have convincingly demonstrated that there is a group of high risk patients whose pregnancies terminated in FL in more than 90% of cases. A personal study of 22 patients showed FL in 50% of LA-positive patients, but after this first FL, subsequent pregnancies also ended up in FL in 23 of 24 cases.[30]

The occurrence of thrombosis in the mother during, or above all, immediately after pregnancy[31–33] is also an argument for screening for APL, and besides, two of three patients in Soulier and Boffa's[3] original description had had a history of thrombosis. However, the association of FL with thrombosis can also be due to a congenital coagulation disorder, especially resistance to activated protein C (APCR). In Brenner et al.,[34] a Leiden mutation of factor V was present in 19 of 39 patients. The problem becomes more difficult in that acquired APCR without a Leiden mutation was present in 9 of 39 patients, and as is well known, APL can induce APCR.[35,36] Apart from thrombosis, the other factors of the APL are also very suggestive (Table 2); early preeclampsia (at approximately 30 weeks gestation) or placental abruption, would also indicate screening for APL.[7]

In the specific case of systemic lupus erythematous (SLE), FL is associated with APL positivity, as demonstrated in particular by Derksen et al.[37] for LA and Deleze et al.[38] for ACL. The prospective study by Petri et al.[39] showed that only second trimester FL appears to be associated with APL positivity. In any case, ACL screening is mandatory in all SLE patients considering pregnancy (in the same way as testing for anti-SSA/SSB antibodies).

Lastly, placental histology may suggest an APL syndrome in the first FL if it demonstrates the

TABLE 2. Clinical manifestations associated with APL

| |
|---|
| Obstetrical |
| Recurrent FL |
| Intrauterine growth retardation |
| Infertility |
| Preeclampsia |
| Placental abruption |
| Vascular |
| Venous thrombosis |
| Arterial thrombosis |
| Neurological |
| Transient cerebral ischemic attacks |
| Stroke |
| Epilepsy |
| Chorea |
| Multi-infarct dementia |
| Cardiac |
| Mitral and aortic valve disease |
| Intracardiac thrombosis |
| Myocardial infarction |
| Cardiomyopathy |
| Dermatological |
| Skin necrosis |
| Livedo reticularis |
| Subungual hemorrhage |
| Leg ulcers |
| Miscellaneous |
| Renal failure |
| Budd-Chiari syndrome |
| Hypertension |
| Pulmonary hypertension |

presence of numerous placental thromboses. Evidence of such thromboses was reported by Nilsson et al.[40] as early as 1975 and was confirmed in subsequent pathological studies.[41–43] Such patients must be screened for APL in the same way as for congenital anomalies (especially APCR).

### WHICH APL IS THE BEST MARKER FOR FL?

In APLS, treponema serology is usually negative (88% of negativity in a personal series of 25 patients); when VDRL gives a false-positive, it is a reliable marker of FL. Thornton et al.[44] estimated the relative risk of FL to be 28.5 in these circumstances.

According to Derksen et al.[37] and Petri and Allbritton,[45] LA is the best marker of FL. Derksen et al.[37] report 75% FL in patients with SLE testing positive for LA vs. 19% in those testing negative. Petri and Allbritton[45] found LA in 40% of lupus patients with FL and 26% of lupus patients achieving full-term pregnancies.

According to other authors,[46] ACL is the best predictive factor for FL, but this finding should be qualified. The risk increases as ACL levels increase.[47,48] Persistence of ACL positivity is particularly important;[49] transitory ACL positivity has practically no diagnostic value.[50] In SLE patients, the relative risk of FL is 10 in ACL-positive patients compared with ACL-negative patients.[38]

Aoki et al.[51] demonstrate that among APLS, the incidence of antibodies to anionic beta 2 GP1-dependent phospholipids appears to be higher in patients who have had FL. However, when testing patients with APLS at risk for thrombosis, anti-beta 2 GP1 appears to be less sensitive than LA. Commercially available kits for global screening of APL also appear to have low sensitivity. In a personal study of 25 patients with ALPS and ACL, the sensitivity of screening with BMD kits was only 76%, and this test did not detect two of ten patients with recurrent FL syndrome. In the event of negative findings with the standard laboratory tests, in the context of highly suggestive clinical findings, screening for antibodies to anti-beta 2 GP1, or to neutral phospholipid such as phosphatidylethanolamine, is justified.[52]

In practice, evaluation of LA and ACL initially and at 8 weeks should be recommended in patients with a suspicion of APLS. In clinically suggestive cases with negative laboratory findings, it is justified to also test for false-positive VDRL, anti-beta 2 GP1, and antiphosphatidylethanolamine.

### MECHANISM OF FL

Additional evidence of the relationship between APL and FL is shown with experimental models.[53] These models lead us to the investigation of the precise mechanism of FL.

1. Pathological findings show numerous placental infarcts in these patients,[40,41,43] suggesting that FL might be caused by thrombosis of the placental vasculature. There is no agreement as to the mechanism causing these thromboses. A fibrinolytic disorder, inhibition of prostacyclin formation by vascular walls,[54] and a disorder of the protein C pathway[55] have successively been mentioned, and, more recently, an interaction between APL and annexin V.[56] The two latter hypotheses are the most convincingly documented.

As far as the protein C pathway is concerned, the presence of an acquired deficit of free protein S appears to be established in both APLS secondary

TABLE 3. Available therapeutic trials: antithrombotic drugs

| | Authors | No. of patients (No. of pregnancies) | Results |
|---|---|---|---|
| Aspirin | Sanchez-Guerrero and Alarcon-Segovia, 1992[66] | 7 (9) | 5 LB[a], 2 FL |
| Heparin | Rosove et al., 1990[23] | 14 (15) | 14 LB, 1 FL |
| Aspirin ± heparin | Kutteh, 1996[24] | 25 aspirin | 44% LB |
| | | 25 aspirin + heparin | 80% LB |
| | Rai et al., 1997[25] | 45 heparin | 42% LB |
| | | 45 aspirin + heparin | 71% LB |
| Omega 3 fatty acids | Rossi and Costa, 1993 | 22 | 21 LB, 1 FL |

[a]LB = live birth.

to SLE and in primary APLS.[57–59] APLS have also been shown to induce an APCR[36] without mutation of factor V, which can be corrected by the addition of platelet extracts.[35] Inhibition of protein C activation by thrombomodulin is much more hypothetical.[55]

Concerning the action of APL on annexin V, Rand et al.[56] have recently compared three patients with recurrent FL to three controls. Levels of annexin V on the surface of trophoblasts were reduced in the presence of IgG APL of patients compared to controls; the coagulation time of plasma in contact with trophoblasts was also longer in the presence of IgG APL patients. This implies that the usual cascade sequence for blood coagulation is physiologically inhibited by the annexin V deposited on the surface of trophoblasts and that APLs interfere with annexin V, preventing it from depositing on the membrane of trophoblasts.

2. In vitro studies suggest that APLs also act on trophoblasts at a very early stage by inhibiting syncytia formation.[60] Murine models have demonstrated that mice immunized by IgG ACL have low fertility and high fetal resorption rates.[61] This accounts for the high rate of first trimester FL and suggests that therapy should be initiated very early, even before fertilization.[62]

### THERAPEUTIC MANAGEMENT

Considerable advances have been made since 1983 when Lubbe et al.,[63] in New Zealand, demonstrated that administration of steroids and low dose aspirin resulted in live births in practically all patients in a small group with a very high risk of FL.

The first question to be addressed is at what point treatment should be initiated, i.e., starting with which pregnancy, and at what time in the pregnancy. If the APLS is diagnosed because of FL, a complete workup, particularly immunological, should be carried out certainly by the third pregnancy, and APL positivity confirmed. In practice, this workup is often initiated after the second FL. Therefore, the patient will initially be treated at her third or fourth pregnancy. It seems logical to start treatment as soon as pregnancy is confirmed, since it has been shown that APL may inhibit the development of trophoblasts, thus inducing very early FL.

If APL positivity has been established before the first pregnancy, for instance in a SLE patient presenting with thrombosis, or even in an asymptomatic woman being operated or before she gets married, and if the patient is given no drug when she gets pregnant, in principle it would be possible to give her no treatment since such a patient is known to have a 1 in 2 chance of a full-term pregnancy despite APL positivity.[30] However, routine administration of aspirin (50–150 mg daily) starting from this first pregnancy seems advisable, since such therapy has been shown to be harmless for both the fetus and the mother.[64,65]

What methods are available? In the 1980s, immunomodulant therapy was favored, but it is currently supplemented with antithrombotic therapy.

### Antithrombotic Drugs

Several studies, some open, some randomized,[66,67] have demonstrated that full-term pregnancies could be achieved with aspirin alone, even in patients with a long history of FL (Table 3). In a study by Silver et al.,[67] the success rate with aspirin alone was 100%, but it should be noted that some patients had suffered only one FL at inclusion. On the other hand, a study of 15 pregnancies in 14 high risk women by Rosove et al.[23] showed a 93% success rate (14 live births in 15 pregnancies) with subcutaneous unfractionated heparin at therapeutic dosage starting from the 10th week of preg-

TABLE 4. Available therapeutic trials: Immunomodulant plus antithrombotic drugs

| | Authors | No. of patients (No. of pregnancies) | Results |
|---|---|---|---|
| Steroids + aspirin | Lubbe et al., 1983[63] | 7 | 6 LB[a], 1 FL |
| | Branch et al., 1985[29] | 8 | 5 LB, 3 FL |
| | Lockshin et al., 1989[74] | 21 | 7 LB, 14 FL |
| | Silveira et al., 1992[73] | 11 (12) | 12 LB, 0 FL |
| | Laskin et al., 1997[75] | 46 placebo | 52% LB |
| | | 42 steroids + aspirin | 60% LB |
| Immunoglobulins + aspirin + heparin | Spinnato et al., 1995[81] | 5 | 5 LB, 0 FL |
| Plasma exchanges | Frampton et al., 1987[82] | 1 | 1 LB |
| | Fucher et al., 1989[83] | 1 | 1 LB |

[a]LB = live birth.

nancy. A posteriori confirmation of the efficacy of heparin was given in 1993 by Inbar et al.[68] in a murine model.

Two recent studies demonstrated that combination therapy with low dose aspirin and heparin was more efficient than aspirin alone. In Kutteh's[24] study of 50 patients, after the diagnosis of pregnancy had been confirmed, heparin was given to 25 patients at therapeutic dosage to the end of pregnancy. Success rate was 44% in the group receiving aspirin alone and 80% in the group receiving aspirin plus heparin. In Rai et al.'s[25] randomized study of 90 patients, low dose heparin (5,000 units) was started only when an ultrasound scan showed evidence of fetal heart activity, and it was stopped if an FL occurred, or otherwise at 34 weeks gestation. Success rate was 42% in the group on aspirin and 71% in the group on aspirin plus heparin. The risk of osteoporosis associated with heparin therapy given to patients during their entire pregnancy should be mentioned,[69–71] and it is worth noting that Rai et al.[25] showed a 5.4% decrease of lumbar bone density postnatally.

Lastly, Italian researchers[72] treated 23 pregnancies in 22 patients with high dose omega 3 fatty acids alone (18 capsules daily, whereas the recommended dosage is 6 daily). The success rate was dramatic: 22 live births in 23 pregnancies (96%). It is unclear whether the antiaggregant action of fish oils alone was involved, or if another mechanism should be considered.

### Immunomodulant Drugs

This treatment aims to reduce APL levels. Steroids and high dose intravenous immunoglobulins (IVIg) have been used successively, and also—anecdotally—plasma exchanges. In fact, these therapies have practically always been combined with antithrombotic drugs, so that their specific efficacy cannot be readily assessed (Table 4).

Lubbe et al.[63] in 1983 and Branch et al.[29] in 1985 must be credited with demonstrating that in small (fewer than 10) groups of patients at very high risk of FL, administration of steroids and aspirin resulted in live births in most cases. Subsequent findings are conflicting. According to Silveira et al.[73] this combination is very efficacious. According to Lockshin et al.,[74] it is not. In Lockshin et al.'s[74] study, 14 FL occurred in 21 pregnancies, but it should be noted that the dosage of steroids was not standardized and it was tapered rapidly during the pregnancy. In a recent randomized study by Laskin et al.,[75] the combination of steroids and aspirin was no better than placebo, but in this study APL-positive patients were mixed with patients testing positive for antinuclear antibodies only. If only APL-positive patients are taken into account, the success rate is 60% in the group receiving steroids and aspirin and 52% in the placebo group. However, it should be noted that the success rate in the placebo group is considerable, while it is admitted that less than 10% full-term pregnancies are achieved in high risk patients.[28,29] In a personal non-randomized series including 27 LA-positive patients who had experienced several FL, 8 patients were given steroids alone and 19 patients steroids plus aspirin. The success rate was 3 of 8 (37.5%) in the group on steroids alone, and 10 of 19 (53%) in the group on steroids plus aspirin. The numbers were too small to reach significance. In this study, LA negativation was a good predictor of favorable outcome (68% live births in the subset of those who became LA-negative patients).

IVIg therapy has been successfully tested in at

TABLE 5. Available therapeutic trials: antithrombotic ± immunomodulant drugs

| | Authors | No. of patients | Results |
|---|---|---|---|
| Aspirin ± steroids | Silver et al., 1993[67] | 22 aspirin | 22 LB[a] 0 FL |
| | | 12 aspirin + steroids | 12 LB, 0 FL |
| Aspirin + steroids or heparin | Cowchock et al., 1992[84] | 8 aspirin + steroids | 6 LB, 2 FL |
| | | 12 aspirin + heparin | 9 LB, 3 FL |

[a]LB = live birth.

least 12 reports in the literature,[76–82] but in all cases aspirin and/or heparin and/or steroids were combined. We have used IVIg in three cases after failure of standard steroid plus aspirin treatment, successfully in all three. In one case, the outcome was satisfactory in spite of increasing ACL and beta 2 GP1 antibodies increasing again at the end of pregnancy.

Two reports of the efficacy of plasma exchanges have been published.[82,83]

### Combination Therapies (Antithrombotic and/or Immunomodulant Drugs)

At least two randomized studies have compared steroids and antithrombotic drugs (Table 5). In the study by Silver et al.[67] mentioned above, the outcome was dramatic in the group on aspirin alone (N = 22) and in the group on aspirin plus steroids (N = 12) since no FL occurred in either group. However, patients could be included after a single FL and 5 of 17 initially randomized patients in the aspirin plus steroids group were not evaluated. A randomized study by Cowchock et al.[84] compared heparin with steroids in a small group of 20 patients who also received aspirin; success rate was 75% in each group. In both studies,[67,84] premature deliveries were more frequent in patients who received steroids.

### Therapeutic Strategies

In women with a history of at least three FL, a minimal immunological workup (antinuclear antibodies, IgG ACL, LA) seems advisable. In practice, this is often done after only two FL. The initial intervention may include aspirin alone, 50–150 mg daily. After failure of this therapy, or if three FL have already occurred and if the patient is relatively old, aspirin and heparin may be prescribed from the beginning of pregnancy. We consider that the choice of a therapeutic dose of heparin administered in two subcutaneous injections is justified. Low molecular weight heparins are not licenced in France during pregnancy, but they have proved to be harmless in studies conducted in France and elsewhere. Laboratory follow-up during pregnancy includes at least a complete blood count (CBC), lactate dehydrogenase (LDH), haptoglobin and reactive protein, creatinine and uric acid, and transaminases (so that a HELLP syndrome is not missed). Fetal ultrasound scan with Doppler studies of the uterines and umbilical arteries are recommended from 22 weeks gestation; Doppler study of the uterine arteries is performed as early as 15 or 16 weeks gestation by some teams. Aspirin should be stopped at 35 or 36 weeks gestation, so that it has been withdrawn for at least 1 week when epidural anesthesia is performed. At that stage, aspirin should be replaced with two daily injections of low molecular weight heparin; if the patient was already on heparin, this treatment should be continued. Heparin should be interrupted for as short a period as possible at the time of delivery. These precautions are mandatory in patients with a history of thrombosis. Strokes have been observed in the immediate postpartum period in patients in whom all antithrombotic drugs had been withdrawn.[32]

In the event of failure of combined aspirin plus heparin therapy, treatment with aspirin plus steroids 0.5 mg/kg/day, or even with aspirin plus steroids plus heparin, should be administered. Should this treatment fail, further treatment may include IVIg, bearing in mind that it implies a course of treatment every 20–30 days to the end of pregnancy.

### REFERENCES

1. Coulam CB: Epidemiology of recurrent spontaneous abortion. Am J Reprod Immunol 26:23–27, 1991.
2. Khamashta MA, Mackworth-Young C: Antiphospholipid (Hughes') syndrome. Br Med J 314:244, 1997.
3. Soulier JP, Boffa MC: Avortements à répétition, thromboses et anticoagulant circulant anti-thromboplastine. Trois observations. Presse Méd 9:859–864, 1980.
4. Harris EN, Bagueley E, Asherson RA, et al.: Clinical and serological features of the antiphospholipid syndrome. Br J Rheum 26:19, 1987.
5. Piette JC, Wechsler B, Blétry O, et al.: Quels critères pour le diagnostic du syndrome des antiphospholipides? Rev Méd Interne 14:799–803, 1993.
6. Reber G, Arvieux J, Comby E, et al.: Multicenter evaluation of nine commercial kits of the quantitation of anticardiolipin antibodies. Thromb Haemostas 73:444–452, 1995.
7. Gleicher N, Pratt D, Dudkiewicz A: What do we really know about autoantibody abnormalities and reproductive failure: A critical review. Autoimmunity 16:115–140, 1993.
8. Branch DW, Andres R, Digre KB, et al.: The association of antiphospholipid antibodies with severe preeclampsia. Obstet Gynecol 73:541–545, 1989.
9. Cervera R, Asherson RA, Font J, et al.: Chorea in the antiphospholipid syndrome: Clinical, radiologic, an immunologic characteristics of 50 patients from our clinics and the recent literature. Medicine 76:203–212, 1997.
10. Branch DW: Thoughts on the mechanism of pregnancy loss associated with the antiphospholipid syndrome. Lupus 3:275–280, 1994.
11. Lockwood CJ, Romero R, Feinberg RF, et al.: The prevalence and biological significance of lupus anticoagulant and anticardiolipin antibodies in a general obstetric population. Am J Obstet Gynecol 161:369–373, 1989.
12. Harris EN, Spinnazo J: Should sera of healthy pregnant women be screened for anticardiolipin antibodies? Arthritis Rheum 33:528, 1990.
13. Parke AL, Wilson D, Maier D: The prevalence of antiphospholipid antibodies in women with recurrent spontaneous abortion, women with successful pregnancies, and women who have never been pregnant. Arthritis Rheum 34:1231–1235, 1991.
14. Lynch A, Marlar R, Murphy J, et al.: Antiphospholipid antibodies in predicting adverse pregnancy outcome. A prospective study. Ann Intern Med 120:470–475, 1994.
15. Infante-Rivard C, David M, Gauthier R, et al.: Lupus anticoagulants, anticardiolipin antibodies, and fetal loss. A case-control study. N Engl J Med 325:1063–1066, 1991.
16. Cowchock S, Smith JB, Gocial B: Antibodies to phospholipids and nuclear antigens in patients with repeated abortions. Am J Obstet Gynecol 155:1902–1910, 1986.
17. Edelman PH, Verdy E, Rouguette AM, et al.: Désordres immunologiques de la coagulation dans la maladie abortive. Etude prospective. Presse Méd 15:961–964, 1986.
18. Howard MA, Firkin BG, Healy DL, et al.: Lupus anticoagulant in women with multiple spontaneous miscarriage. Am J Hematol 26:175–178, 1987.
19. Unander AM, Norberg R, Hahn L, et al.: Anticardiolipin antibodies and complement in ninety-nine women with habitual abortion. Am J Obstet Gynecol 156:114–119, 1987.
20. Creagh MD, Malia RG, Cooper SM, et al.: Screening for lupus anticoagulant and anticardiolipin antibodies in women with fetal loss. J Clin Pathol 44:45–47, 1991.
21. Petri M, Golbus M, Anderson R, et al.: Antinuclear antibody, lupus anticoagulant, and anticardiolipin antibody in women with idiopathic habitual abortion. A controlled, prospective study of forty-four women. Arthritis Rheum 30:601–606, 1987.
22. Branch DW, Silver RM, Blackwell JL, et al.: Outcome of treated pregnancies in women with antiphospholipid syndrome: An update of the Utah experience. Obstet Gynecol 80:614–620, 1992.
23. Rosove MH, Tabsh K, Wasserstrum N, et al.: Heparin therapy for pregnant women with lupus anticoagulant or anticardiolipin antibodies. Obstet Gynecol 75:630–634, 1990.
24. Kutteh WH: Antiphospholipid antibody-associated recurrent pregnancy loss: Treatment with heparin and low-dose aspirin is superior to low-dose aspirin alone. Am J Obstet Gynecol 174:1584–1589, 1996.
25. Rai R, Cohen H, Dave M, et al.: Randomised controlled trial of aspirin and aspirin plus heparin in pregnant women with recurrent miscarriage associated with phospholipid antibodies (or antiphospholipid antibodies). Br Med J 314:253–257, 1997.
26. Muller-Eckhardt G, Heine O, Polten B: IVIG to prevent recurrent spontaneous abortion. Lancet 337:424, 1991.
27. Kwak FMY, Kwak JYH, Becr AE: Peripheral blood natural killer cells are effectively suppressed by immunoglobulin G infusion in women with recurrent spontaneous abortion. Am J Reprod Immunol 33:466–470, 1995.
28. Lubbe WF, Liggins GC: Lupus anticoagulant, thrombosis and pregnancy. Am J Obstet Gynecol 153:322–327, 1985.
29. Branch DW, Scott JR, Kochenour NK, et al.: Obstretric complications associated with the lupus anticoagulant. N Engl J Med 313:1322–1326, 1985.
30. Godeau P, Blétry O, Piette JC, et al.: Anticoagulants circulants, conditions cliniques du diagnostic. Rev Méd Interne 6:523–541, 1985.
31. Thorp JM, Chescheir NC, Fann B: Postpartum myocardial infarction in a patient with antiphospholipid syndrome. Am J Perinatol 11:1–3, 1994.
32. Le Thi Huong DU, Wechsler B, Edelman P, et al.: Postpartum cerebral infarction associated with aspirin withdrawal in the antiphospholipid antibody syndrome. J Rheumatol 20:1229–1232, 1993.
33. Kupfermine MJ, Lee MJ, Green D, et al.: Severe postpartum pulmonary, cardiac, and renal syndrome associated with antiphospholipid antibodies. Obstet Gynecol 83:806–807, 1994.
34. Brenner B, Mandel H, Lanir N, et al.: Activated protein C resistance can be associated with recurrent fetal loss. Br J Haematol 97:551–554, 1997.
35. Martorell JR, Munoz-Castillo A, Gil JL: False positive activated protein C resistance test due to antiphospholipid antibodies is corrected by platelet extract. Thromb Haemostas 74:796–797, 1995.
36. Ehrenforth S, Radtke KR, Scharrer I: Acquired activated protein C-resistance in patients with lupus anticoagulants. Thromb Haemostas 74:797–798, 1995.
37. Derksen RHWM, Bouma BN, Kater L: The striking association between lupus anticoagulant and fetal loss in systemic lupus erythematosus patients. Arthritis Rheum 29:695–696, 1986.
38. Deleze M, Alarcon-Segovia D, Valdes-Macho E, et al.: Relationship between antiphospholipid antibodies and recurrent fetal loss in patients with systemic lupus erythematosus and apparently healthy women. J Rheumatol 16:768–772, 1989.
39. Petri M, Howard D, Repke J, et al.: The Hopkins lupus pregnancy center: 1987–1991 update. Am J Reprod Immunol 28:188–191, 1992.
40. Nilsson IM, Astedt B, Hedner U, et al.: Intrauterine death and circulating anticoagulant ("antithromboplastin"). Acta Med Scand 197:153–159, 1975.
41. De Wolf F, Carreras LO, Moerman P, et al.: Decidual vasculopathy and extensive placental infarction in a patient with repeated thromboembolic accidents, recurrent fetal loss, and a lupus anticoagulant. Am J Obstet Gynecol 142:829–834, 1982.
42. Hanly JG, Gladman DD, Rose TH, et al.: Lupus pregnancy. A prospective study of placental changes. Arthritis Rheum 31:358–367, 1988.
43. Out HJ, Kooijman CD, Bruinse HW, Derksen RH: Histopathological findings in placentae from patients with intra-uterine fetal death and anti-phospholipid antibodies. Eur J Obstet Gynecol Reprod Biol 41:179–186, 1991.
44. Thornton JG, Foote GA, Page CE, et al.: False positive results of tests for syphilis and outcome of pregnancy: A retrospective case-control study. Br Med J 295:355–356, 1987.
45. Petri M, Allbritton J: Fetal outcome of lupus pregnancy: A retrospective case-control study of the Hopkins lupus cohort. J Rheumatol 20:650–656, 1993.
46. Lockshin MD, Qamar T, Druzin ML, et al.: Antibody to cardiolipin, lupus anticoagulant, and fetal death. J Rheumatol 14:259–262, 1987.
47. Loizou S, Byron MA, Englert HJ, et al.: Association of quantitative anticardiolipin antibody levels with fetal loss and time of loss in systemic lupus erythematosus. Am J Med 68:525–531, 1988.
48. Harris EN, Chan JKH, Asherson RA, et al.: Thrombosis, recurrent fetal loss, and thrombocytopenia; predictive value of the anticardiolipin antibody test. Arch Intern Med 146:2153–2156, 1986.
49. Alarcon-Segovia D, Deleze M, Oria CV, et al.: Antiphospholipid antibodies and the antiphospholipid syndrome in systemic lupus erythematosus. Medicine 68:353–363, 1989.
50. Ginsberg JS, Brill-Edwards P, Johnston M, et al.: Relationship of antiphospholipid antibodies to pregnancy loss in patients with systemic lupus erythematosus: A cross-sectional study. Blood 80:975–980, 1992.
51. Aoki K, Dudkiewicz AB, Matsuura E, et al.: Clinical significance of beta 2 glycoprotein 1-dependent anticardiolipin antibodies in the reproductive autoimmune failure syndrome: Correlation with conventional antiphospholipid antibody detection systems. Am J Obstet Gynecol 172:926–931, 1995.
52. Rote MS, Dostal-Johnson D, Branch WD: Antiphospholipid antibodies and recurrent pregnancy loss: Correlation between the activated partial thromboplastin time and antibodies against phosphatidylserine and cardiolipin. Am J Obstet Gynecol 163:575–584, 1990.
53. Branch DW, Dusley DJ, Mitchell MD, et al.: Immunoglobulin G fractions from patients with antiphospholipid antibodies cause fetal death in BALB/c mice: A model for autoimmune fetal loss. Am J Obstet Gynecol 163:210–216, 1990.
54. Carreras LO, Vermylen JG: "Lupus" anticoagulant and thrombosis—Possible role of inhibition of prostacyclin formation. Thromb Haemostas 48:38–40, 1982.
55. De Groot PG, Derksen RHWM: Protein C pathway, antiphospholipid antibodies and thrombosis. Lupus 3: 229–233, 1994.
56. Rand JH, Wu XX, Andree HAM, et al.: Pregnancy loss in the antiphospholipid-antibody syndrome: A possible thrombogenic mechanism. N Engl J Med 337:154–160, 1997.
57. Ginsberg JS, Demers C, Brill-Edwards P, et al.: Acquired free protein S deficiency is associated with antiphospholipid antibodies and increased thrombin generation in patients with systemic lupus erythematosus. Am J Med 98:379–383, 1995.
58. Crowther MA, Johnston M, Weitz, et al.: Free protein S deficiency may be found in patients with antiphospholipid antibodies who do not have systemic lupus erythematosus. Thromb Haemostas 76:689–691, 1996.
59. Bokarewa MI, Bremme K, Falk G, et al.: Studies on phospholipid antibodies, APC resistance and associated mutation in the coagulation factor V gene. Thromb Res 78:193–200, 1995.
60. Lyden TW, Vogt E, AK Ng, et al.: Monoclonal antiphospholipid antibody reactivity against human placental trophoblast. J Reprod Immunol 22:1–14, 1992.
61. Sthoeger ZM, Mozes E, Tartakovsky B: Anticardiolipin antibodies induce pregnancy failure by impairing embryonic implantation. Proc Natl Acad Sci USA 90:6464–6467, 1993.
62. Sher G, Feinman M, Zouves C, et al.: High fecundity rates following in vitro fertilization and embryo transfer in antiphospholipid antibody seropositive women treated with heparin an aspirin. Hum Reprod 9:2278–2283, 1994.
63. Lubbe WF, Butler WS, Palmer SJ, et al.: Fetal survival after prednisone suppression of maternal lupus anticoagulant. Lancet 1:1361–1363, 1983.
64. Uzan S, Beaufils M, Bréart G, et al.: Prevention of fetal growth retardation with low-dose aspirin: Findings of the EPREDA trial. Lancet 337:1427–1431, 1991.
65. Sibaï BM, Caritis SN, Thom E, et al.: Prevention of preeclampsia with low-dose aspirin in healthy, nulliparous pregnant women. N Engl J Med 329:1213–1218, 1993.
66. Sanchez-Guerrero J, Alarcon-Segovia D: Course of antiphospholipid antibodies in patients with primary antiphospholipid syndrome before, during, and after pregnancy treated with low dose aspirin. Relationship of antibody levels to outcome in 7 patients. J Rheumatol 19:1083–1088, 1992.
67. Silver RK, MacGregor SN, Sholl JS, et al.: Comparative trial of prednisone plus aspirin versus aspirin alone in the treatment of anticardiolipin antibody-positive obstetric patients. Am J Obstet Gynecol 169:1411–1417, 1993.
68. Inbar O, Blank M, Faden D, et al.: Prevention of fetal loss in experimental antiphospholipid syndrome by low-molecular-weight heparin. Am J Obstet Gynecol 169: 423–426, 1993.
69. Wise PH, Hall AJ: Heparin-induced osteopenia in pregnancy. Br Med J 281:110–111, 1980.
70. Dahlman T, Lindvall N, Hellgren M: Osteopenia in pregnancy during long-term heparin treatment: A radiological study post partum. Br J Obstet Gynaecol 97:221–228, 1990.
71. Douketis JD, Ginsberg JS, Burrows RF: The effects of long-term heparin therapy during pregnancy on bone density. Thromb Haemostas 75:254–257, 1996.
72. Rossi E, Costa M: Fish oil derivatives as a prophylaxis of recurrent miscarriage associated with antiphospholipid antibodies: A pilot study. Lupus 2:319–323, 1993.
73. Silveira LH, Hubble CI, Jara LJ, et al.: Prevention of anticardiolipin antibody-related pregnancy loss with prednisone and aspirin. Am J Med 93:403–411, 1992.
74. Lockshin MD, Druzin ML, Qamar T: Prednisone does not prevent recurrent fetal death in women with antiphospholipid antibody. Am J Obstet Gynecol 160:439–443, 1989.
75. Laskin CA, Bombardier C, Hannah ME, et al.: Prednisone and aspirin in women with autoantibodies and unexplained recurrent fetal loss. N Engl J Med 337:148–153, 1997.
76. Carreras LO, Perez GN, Vega HR, Casavilla F: Lupus anticoagulant and recurrent fetal loss: Successful treatment with gammaglobulin. Lancet 2:393–394, 1988.
77. Scott JR, Branch DW, Kochenour NK, et al.: Intravenous immunoglobulin treatment of pregnant patients with recurrent pregnancy loss caused by antiphospholipid antibodies and Rh immunization. Am J Obstet Gynecol 159:1055–1056, 1989.
78. François A, Freund M, Daffos F, et al.: Repeated fetal losses and the lupus anticoagulant. Ann Intern Med 109: 993–994, 1988.
79. Parke A, Maier D, Wilson D, et al.: Intravenous gammaglobulin, antiphospholipid antibodies, and pregnancy. Ann Intern Med 110:495–496, 1989.
80. Wapner RJ, Cowchock FS, Shapiro SS: Successful treatment in two women with antiphospholipid antibodies and refractory pregnancy losses with intravenous immunoglobulin infusions. Am J Obstet Gynecol 161:1271–1272, 1989.
81. Spinnato JA, Clark AL, Pierangeli SS, et al.: Intravenous immunoglobulin therapy for the antiphospholipid syndrome in pregnancy. Am J Obstet Gynecol 172:690–694, 1995.
82. Frampton G, Cameron JS, Thom M, et al.: Successful removal of antiphospholipid antibody during pregnancy using plasma exchange and low-dose prednisolone. Lancet 1:1023–1024, 1987.
83. Flucher D, Stewart G, Exnert T, et al.: Plasma exchange and the anticardiolipin syndrome in pregnancy. Lancet 2:171, 1989.
84. Cowchock FS, Reece EA, Balaban D, et al.: Repeated fetal losses associated with antiphospholipid antibodies: A collaborative randomized trial comparing prednisone with low-dose heparin treatment. Am J Obstet Gynecol 166:1318–1323, 1992.
